# Systematic review and meta-analysis of the associations of vegan and vegetarian diets with inflammatory biomarkers

**DOI:** 10.1038/s41598-020-78426-8

**Published:** 2020-12-10

**Authors:** Juliane Menzel, Afraa Jabakhanji, Ronald Biemann, Knut Mai, Klaus Abraham, Cornelia Weikert

**Affiliations:** 1 Department of Food Safety, German Federal Institute for Risk Assessment, Max-Dohrn-Str. 8-10, 10589 Berlin, Germany; 2 Institute for Clinical Chemistry and Pathobiochemistry, Otto-Von-Guericke University Magdeburg, Magdeburg, Germany; 3 Institute of Laboratory Medicine, Clinical Chemistry and Molecular Diagnostics, University of Leipzig, Leipzig, Germany; 4 Department of Endocrinology and Metabolism, Charité - Universitätsmedizin Berlin, Berlin, Germany; 5 Center for Cardiovascular Research (CCR), Charité - Universitätsmedizin Berlin, Berlin, Germany; 6 German Centre for Cardiovascular Research (DZHK), Partner Site Berlin, Berlin, Germany

**Keywords:** Biomarkers, Nutrition, Public health

## Abstract

Plant-based diets like vegetarian or vegan diets might influence circulating levels of inflammatory biomarkers, thereby reducing the risk of chronic diseases. This systematic review and meta-analysis aimed to investigate the associations of veganism and vegetarianism with circulating inflammatory biomarkers in comparison to omnivores. Literature search was conducted in Pubmed and EMBASE until April 2020 and mean differences of biomarkers were assessed for: C-reactive protein (CRP), interleukin-6 (IL-6), interleukin-18 (IL-18), interleukin-1 receptor antagonist (IL-1 RA), tumor necrosis factor-alpha (TNF-ɑ), E-selectin, intercellular adhesion molecule-1 (ICAM-1), monocyte chemoattractant protein-1 (MCP-1), adiponectin, omentin-1 and resistin. Of initially identified 1073 publications, 21 cross-sectional studies met the inclusion criteria and were included in the systematic review and meta-analysis. Vegan diet was associated with lower levels of CRP compared to omnivores [mean difference − 0.54 mg/l, 95%-CI: − 0.79 to − 0.28, *p* < 0.0001]. This association was less pronounced in vegetarians [mean difference − 0.25 mg/l, 95%-CI: − 0.49 to 0.00, *p* = 0.05]. In patients with impaired kidney function, the association between vegetarian nutrition and CRP was much stronger with − 3.91 mg/l (95%-CI: − 5.23 to − 2.60; *p* < 0.0001). No substantial effects were observed for all other inflammatory biomarkers. Despite strong associations between CRP and a vegan or vegetarian diet were seen, further research is needed, as most inflammatory biomarkers were investigated only in single studies so far.

## Introduction

Since recent years, a growing trend for vegetarian and vegan diets can be recognized in Germany and other Western countries^1^. These plant-based diets were typically characterized by a higher consumption of fruits, vegetables, legumes, whole-grains, nuts, and various soy products^2^, corresponding to a lower intake of saturated fat and cholesterol^2^, as well as a larger amounts of antioxidant micronutrients like vitamins C and E, dietary fibre and phytochemicals^2,3^. Due to the increased awareness of environmental problems and compassion for animals, a growing trend toward veganism has remarkably emerged in the past few years. Another decisive factor for people turning to a vegan diet is the potential of health benefits. As a matter of fact, scientific evidence leads to the assumption that a vegetarian or vegan nutrition may be protective against many chronic inflammatory diseases like type 2 diabetes^4^, cardiovascular diseases^5^, or cancer^6^. Interestingly, recent research suggested associations between low-grade inflammation and increased risk of various chronic diseases. Hence, the development of chronic diseases could be influenced by inflammatory biomarkers acting as intermediate risk factors. In fact, it has been found that elevated concentrations of inflammatory markers like high-sensitivity C-reactive protein (hs-CRP), interleukin 6 (IL-6) or tumor necrosis factor-α (TNF-α) were associated with pathogenetic mechanisms of numerous chronic diseases in type 2 diabetes^7^, cardiovascular disease^8^ or selected cancer types^9^. On the contrary, concentrations of adiponectin were inversely associated with these diseases^10–12^.

Recent scientific evidence suggested that plant-based diets may modulate inflammatory biomarker profiles, showing an attenuation of inflammation markers as for instance CRP, IL-6 and soluble intercellular adhesion molecule 1 (sICAM-1)^13–15^. Up to date, meta-analyses noticed that vegetarian nutrition is associated with lower CRP concentrations^3,16^, while the impact of an exclusive vegan diet on inflammatory biomarkers was only examined by few studies. Therefore, we aimed to conduct a systematical review and meta-analysis to investigate the effects of a vegetarian or vegan diet on a comprehensive spectrum of inflammatory biomarkers, i.e. C-reactive protein (CRP), interleukin-6 (IL-6), interleukin-18 (IL-18), interleukin-1 receptor antagonist (IL-1 RA), tumor necrosis factor-alpha (TNF-ɑ), E-selectin, intercellular adhesion molecule-1 (ICAM-1), monocyte chemoattractant protein-1 (MCP-1), adiponectin, omentin-1 and resistin, in healthy and diseased population, separately.

## Results

**Figure 1:**
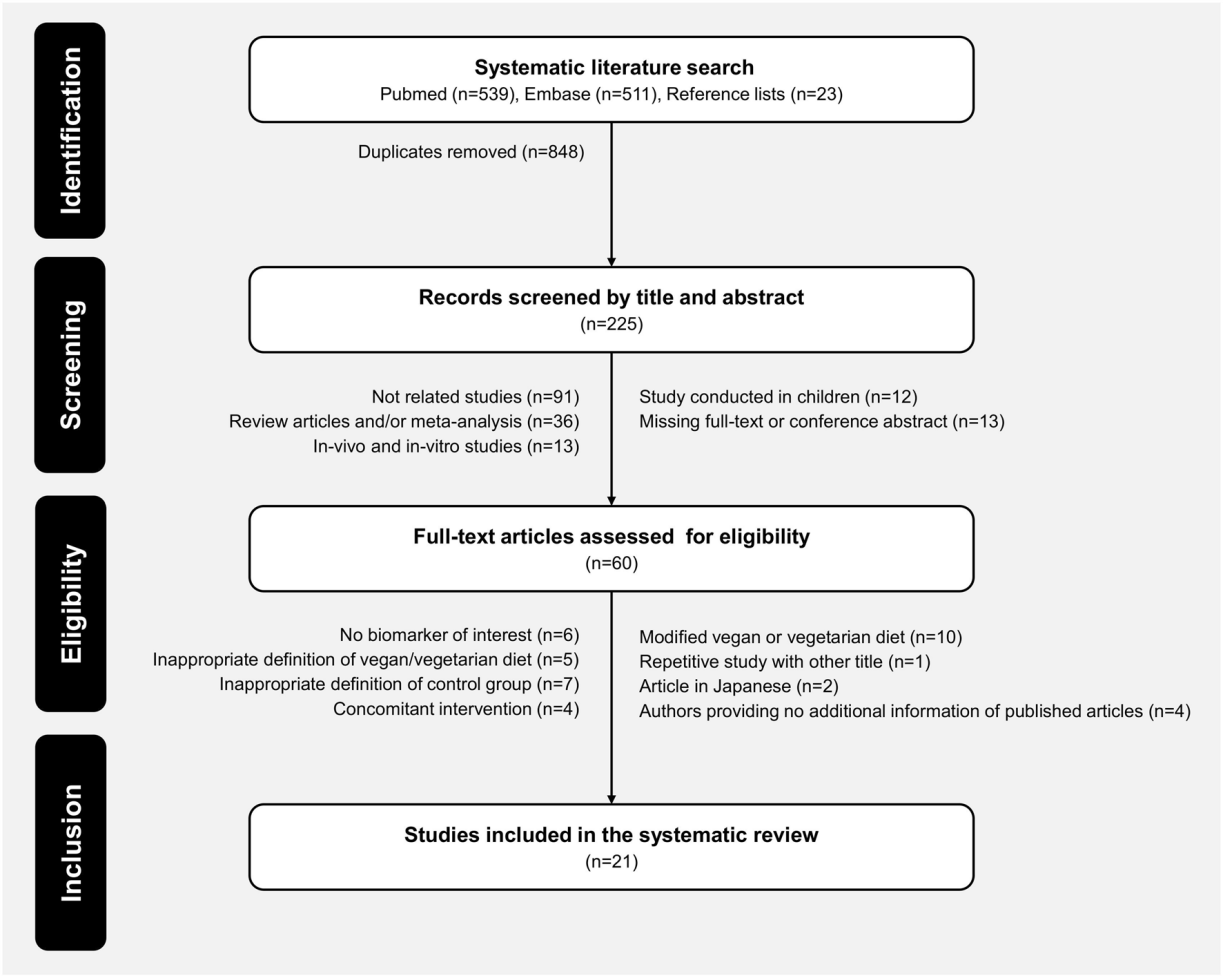
Flowchart. Selection process from initial search to final number of the included studies.

The systematic literature search produced a total of 1073 citations through database searching. In detail, we identified 511 publications from Embase, 539 from PubMed and 23 additional publications from manual searching the reference lists. After exclusion of duplicates from different databases (n = 848), publications reduced to 225 articles. After initial screening by titles and abstracts, 165 publications did not meet the study inclusion criteria and were excluded. From the remaining 60 citations, further 39 records were excluded following full-text assessment. In total, 21 studies have been included in the present systematic review. Detailed processes of study selection are shown in Fig. 1^17–37^.

Main characteristics of the included 21 studies are summarized in Table 1. Studies were conducted across 3 continents, most studies were conducted in Asia (12 studies^19,22–27,29,32,35,36^), followed by Europe (6 studies^18,20,21,28,30,37^) and South America (3 studies^17,33,34^). All studies, with exception of one prospective study^29^, used a cross-sectional design. However, the study with a prospective design has been considered as cross-sectional study, because we only used cross-sectional data from the baseline characteristics.

**Table 1:** Characteristics of reviewed studies on the association of vegan or vegetarian diet with inflammatory biomarkers.

Study characteristics			Diet group characteristics			Studied	SQS
			Vegetarians	Vegans	Omnivores	biomarkers	
**Mezzano et al. (1999)**^17^							
Country	Chile	n	(n = 26)		(n = 26)	CRP	5
Study design	Cross-sectional	Age^a^	47.0 ± 12.7^c^		39.5 ± 12.2^c^		
Study population (n)	Apparently healthy (n = 52)	BMI^b^	23.0 ± 3.3^ci^		23.0 ± 3.3^ci^		
Male % (n)	46.1% (n = 24)	Duration^a^	≥ 1				
**Šebeková et al. (2001)**^18^							
Country	Slovak Republic	n	(n = 19)	(n = 9)	(n = 19)	CRP	5
Study design	Cross-sectional	Age^a^	36.1 ± 2.5^d^	36.6 ± 3.0^d^	30.5 ± 1.6^d^		
Study population (n)	Apparently healthy (n = 61)	BMI^b^	22.0 ± 0.05^d^	20.6 ± 0.8^d^	23.8 ± 0.04^d^		
Male % (n)	38% (n = 23)	Duration^a^	8.2 ± 0.8^c^	7.2 ± 1.0^c^			
**Szeto et al. (2004)**^19^							
Country	China	n	(n = 30)		(n = 30)	CRP	3
Study design	Cross-sectional	Age^a^	44.2 ± 9.0^c^		44.0 ± 9.2^c^		
Study population (n)	Apparently healthy (n = 60)	BMI^b^	NR		NR		
Male % (n)	10% (n = 6)	Duration^a^	21.8 ± 12.2^c^				
**Krajcovicova-Kudlackova et al. (2005)**^20^							
Country	Slovak Republic	n	(n = 133)		(n = 137)	hs-CRP	6
Study design	Cross-sectional	Age^a^	46.2 ± 1.4^d^		47.2 ± 1.4^d^		
Study population (n)	Apparently healthy (n = 270)	BMI^b^	22.9 ± 0.2^e^		24.7 ± 0.3^e^		
Male % (n)	35.5% (n = 96)	Duration^a^	10.4 ± 0.4^c^				
**Šebeková et al. (2006)**^21^							
Country	Slovak Republic	n	(n = 90)		(n = 46)	hs-CRP	5
Study design	Cross-sectional	Age^a^	37.7 (35.1–40.3)^f^		37.7 (33.5–40.7)^f^		
Study population (n)	Apparently healthy (n = 136)	BMI^b^	22.7 (22.1–23.3)^f^		23.8 (22.7–24.9)^f^		
Male % (n)	36% (n = 49)	Duration^a^	2–25				
**Chen et al. (2008)**^22^							
Country	Taiwan	n	(n = 99)		(n = 99)	hs-CRP	5
Study design	Cross-sectional	Age^a^	51.2 ± 8.9^c^		49.4 ± 9.6^c^		
Study population (n)	Apparently healthy (n = 198)	BMI^b^	22.9 ± 2.8^c^		23.8 ± 3.6^c^		
Male % (n)	43.9% (n = 87)	Duration^a^	≥ 1				
**Hung et al. (2008)**^23^							
Country	Taiwan	n	(n = 71)		(n = 388)	CRP	5
Study design	Cross-sectional	Age^a^	49.1 ± 11.2^c^		50.6 ± 9.5^c^		
Study population (n)	Apparently healthy or metabolic syndrome (n = 459)	BMI^b^	22.6 ± 2.7^c^		23.9 ± 3.1^c^		
Male % (n)	57.7% (n = 265)	Duration^a^	NR				
**Chen et al. (2011)**^24^							
Country	Taiwan	n	(n = 173)		(n = 190)	hs-CRP	6
Study design	Cross-sectional	Age^a^	54.0 ± 9.7^c^		49.9 ± 9.8^c^		
Study population (n)	Apparently healthy (n = 363)	BMI^b^	22.9 ± 2.9^c^		23.3 ± 3.5^c^		
Male % (n)	0% (n = 0)	Duration^a^	≥ 1				
**Su et al. (2011)**^25^							
Country	Taiwan	n	(n = 49)		(n = 41)	hs-CRP	3
Study design	Cross-sectional	Age^a^	58.6 ± 6.0^c^		57.2 ± 5.4^c^		
Study population (n)	Apparently healthy (n = 90)	BMI^b^	23.2 ± 2.7^c^		23.1 ± 2.9^c^		
Male % (n)	0% (n = 0)	Duration^a^	10.8 ± 7.5^c^				
**Wu et al. (2011)**^26^							
Country	Taiwan	n	(n = 19)		(n = 299)	hs-CRP	5
Study design	Cross-sectional	Age^a^	63.3 ± 2.6^c^		57.5 ± 1.2^c^		
Study population (n)	Haemodialysis patients (n = 318)	BMI^b^	22.7 ± 0.3^c^		20.2 ± 0.8^c^		
Male % (n)	46.9% (n = 149)	Duration^a^	NR				
**Lee et al. (2014)**^27^							
Country	Republic of Korea	n	(n = 357)		(n = 357)	hs-CRP	6
Study design	Cross-sectional	Age^a^	52.8 ± 8.5^c^		53.8 ± 8.7^c^		
Study population (n)	Apparently healthy or metabolic syndrome (n = 714)	BMI^b^	24.9 ± 2.9^c^		23.7 ± 3.3^c^		
Male % (n)	42.9% (n = 306)	Duration^a^	NR				
**Montalcini et al. (2015)**^28^							
Country	Italy	n	(n = 26)		(n = 26)	IL-6	5
Study design	Cross-sectional	Age^a^	32.6 ± 8.4^c^		30.5 ± 6.7^c^	TNF-α	
Study population (n)	Apparently healthy (n = 52)	BMI^b^	21.9 ± 2.0^c^		21.8 ± 2.0^c^	MCP-1	
Male % (n)	50% (n = 26)	Duration^a^	≥ 3				
**Chuang et al. (2016)**^29^							
Country	Taiwan	n	(n = 686)		(n = 3423)	CRP	7
Study design	Cross-sectional^h^	Age^a^	42.2 ± 12.3^c^		45.1 ± 12.2^c^		
Study population (n)	Apparently healthy (n = 4109)	BMI^b^	22.1 ± 3.1^c^		22.9 ± 3.2^c^		
Male % (n)	27.1% (n = 1115)	Duration^a^	≥ 3				
**Kandouz et al. (2016)**^30^							
Country	United Kingdom	n	(n = 16)		(n = 122)	CRP	5
Study design	Cross-sectional	Age^a^	71.7 ± 11.6^c^		63.7 ± 16.9^c^		
Study population (n)	Kidney failure (n = 138)	BMI^b^	25.4 ± 5.2^c^		26.5 ± 6.0^c^		
Male % (n)	63.9% (n = 88)	Duration^a^	early adult hood				
**Lee et al. (2016)**^31^							
Country	Taiwan	n	(n = 54)		(n = 100)	hs-CRP	4
Study design	Cross-sectional	Age^a^	65.1 ± 11.3^c^		57.7 ± 10.5^c^	IL-6	
Study population (n)	Type 2 diabetes (n = 154)	BMI^b^	24.9 ± 6.2^c^		26.5 ± 5.7^c^		
Male % (n)	41.5% (n = 64)	Duration^a^	≥ 1				
**Ou et al. (2016)**^32^							
Country	Taiwan	n	(n = 21)		(n = 42)	hs-CRP	4
Study design	Cross-sectional	Age^a^	56.2 ± 13.7^c^		56.3 ± 11.7^c^		
Study population (n)	Dialysis Patients (n = 63)	BMI^b^	20.4 ± 2.1^c^		22.5 ± 3.4^c^		
Male % (n)	22.6% (n = 12)	Duration^a^	≥ 1.5				
**Acosta-Navarro et al. (2017)**^33^							
Country	Brazil	n	(n = 44)		(n = 44)	hs-CRP	6
Study design	Cross-sectional	Age^a^	45.5 ± 7.8^c^		46.8 ± 9.6^c^		
Study population (n)	Apparently healthy (n = 88)	BMI^b^	23.1 ± 2.9^c^		27.2 ± 4.8^c^		
Male % (n)	100% (n = 88)	Duration^a^	17.8 ± 12.5^c^				
**Franco-De-Moraes et al. (2017)**^34^							
Country	Brazil	n	(n = 102)	(n = 66)	(n = 100)	CRP	5
Study design	Cross-sectional	Age^a^	49.6 ± 8.6^c^	49.6 ± 8.5^c^	49.1 ± 8.2^c^	E-selectin	
Study population (n)	Apparently healthy (n = 268)	BMI^b^	24.2 ± 3.9^c^	23.2 ± 4.1^c^	26.4 ± 4.7^c^	TNF-α	
Male % (n)	45.8% (n = 123)	Duration^a^	≥ 1	≥ 1			
**Tseng et al. (2018)**^35^							
Country	Taiwan	n	(n = 15)		(n = 140)	hs-CRP	5
Study design	Cross-sectional	Age^a^	63.2 ± 2.5^c^		58.5 ± 1.3^c^		
Study population (n)	Haemodialysis patients (n = 155)	BMI^b^	20.2 ± 0.5^c^		22.4 ± 0.2^c^		
Male % (n)	45.1% (n = 70)	Duration^a^	NR				
**Ganie et al. (2019)**^36^							
Country	India	n	(n = 179)		(n = 141)	hs-CRP	7
Study design	Cross-sectional	Age^a^	26.5 ± 6.0^c^		26.6 ± 4.1^c^	IL-6	
Study population (n)	Apparently healthy (n = 320)	BMI^b^	24.0 ± 4.2^c^		24.0 ± 3.6^c^	TNF-α	
Male % (n)	0% (n = 0)	Duration^a^	NR			Adiponectin	
Study population (n)	PCOS (n = 144)	n	(n = 82)		(n = 62)	Resistin	
Male % (n)	0% (n = 0)	Age^a^	25.7 ± 3.8^c^		26.1 ± 4.4^c^		
		BMI^b^	24.9 ± 3.6^c^		24.6 ± 3.5^c^		
		Duration^a^	NR				
**Menzel et al.(2020)**^ 37^							
Country	Germany	n		(n = 36)	(n = 36)	hs-CRP	6
Study design	Cross-sectional	Age^a^		37.5 (32.5–44.0)^g^	38.5 (32.0–46.0)^g^	IL-18	
Study population (n)	Apparently healthy (n = 72)	BMI^b^		22.9 ± 3.2^c^	24.0 ± 2.1^c^	IL-1 RA	
Male % (n)	50% (n = 36)	Duration^a^		4.8 (3.1–8.7)^g^		ICAM-1	
						Adiponectin	
						Omentin-1	
						Resistin	

^a^Reported in years; ^b^ reported in kg/m^2^; ^c^expressed as mean ± SD; ^d^mean ± SE; ^e^mean ± SEM; ^f^ mean (95%-CI); ^g^ median (IQR); ^h^ using baseline values; ^i^ mean across both diet groups; SQS: Study Quality Score (using Newcastle—Ottawa Quality Assessment Scale adapted for cross-sectional studies); NR: not reported.

Twenty studies investigated the association between a vegetarian diet and inflammatory biomarkers compared to omnivores^17–36^, two studies of them examined also the association between a vegan diet and inflammatory biomarkers, in parallel to a vegetarian diet^18,34^. The study by Menzel et al.^37^ investigated the association between a vegan diet and inflammatory biomarkers compared to an omnivorous diet only. In total, the duration time following a vegan diet ranged from at least one year up to 20 years, and for vegetarian diet from one year up to 25 years.

Selected studies were published between 1999^17^ to 2020^37^. Numbers of study participants varied between n = 52^17,28^ and n = 4109^29^. Overall, analysis comprised a total number of 2291 vegetarians, 111 vegans and 5868 omnivores, with a mean age of 46.2 years (vegans: 41.7 years; vegetarians: 49.6 years; omnivores: 47.2 years). The BMI was on average 23.1 kg/m^2^ of all studies (not reported for Szeto et al.^19^) (vegans: 22.2 kg/m^2^; vegetarians: 23.0 kg/m^2^; omnivores: 23.9 kg/m^2^). Of all participants, 89.8% had a healthy mean BMI of < 25.0 kg/m^2^ and 10.2% had a mean BMI between 25.0 and 29.9 kg/m^2^. No study included participants with mean BMI ≥ 30 kg/m^2^.

The majority of the studies were conducted in apparently healthy participants comprising 88.2% of the participants (16 out of 21 studies, Table 1). Of note, Ganie et al.^36^ investigated 320 healthy participants and 144 women with polycystic ovary syndrome (PCOS), both groups separated by diet (vegetarian vs. non-vegetarian). Hung et al.^23^ and Lee et al.^27^ conducted the study in a mixed population of apparently healthy participants and patients with metabolic syndrome (less than 20% of the study population). Moreover, six studies were conducted in participants diagnosed with impaired kidney function^26,30,32,35^ , type 2 diabetes^31^ or PCOS^36^. Seventeen studies used populations consisting of men and women, only Acosta-Navarra et al.^33^ included exclusively male individuals, and three other studies included only female subjects^24,25,36^. In total, 38.2% of all involved participants were male. With regard to the outcome assessment, most of the studies focused on CRP (n = 20^17–27,29–37^), whereas a restricted number of studies evaluated IL-6 (n = 3^28,31,36^), TNF-ɑ (n = 3^28,34,36^), adiponectin (n = 2^36,37^) or resistin (n = 2^36,37^). The other inflammatory biomarkers E-selectin (n = 1^34^), MCP-1 (n = 1^28^), IL-18, IL-1 RA, ICAM-1 and omentin-1 were only analyzed in one study^37^.

### Association of vegan diet and inflammatory biomarkers

**Figure 2:**
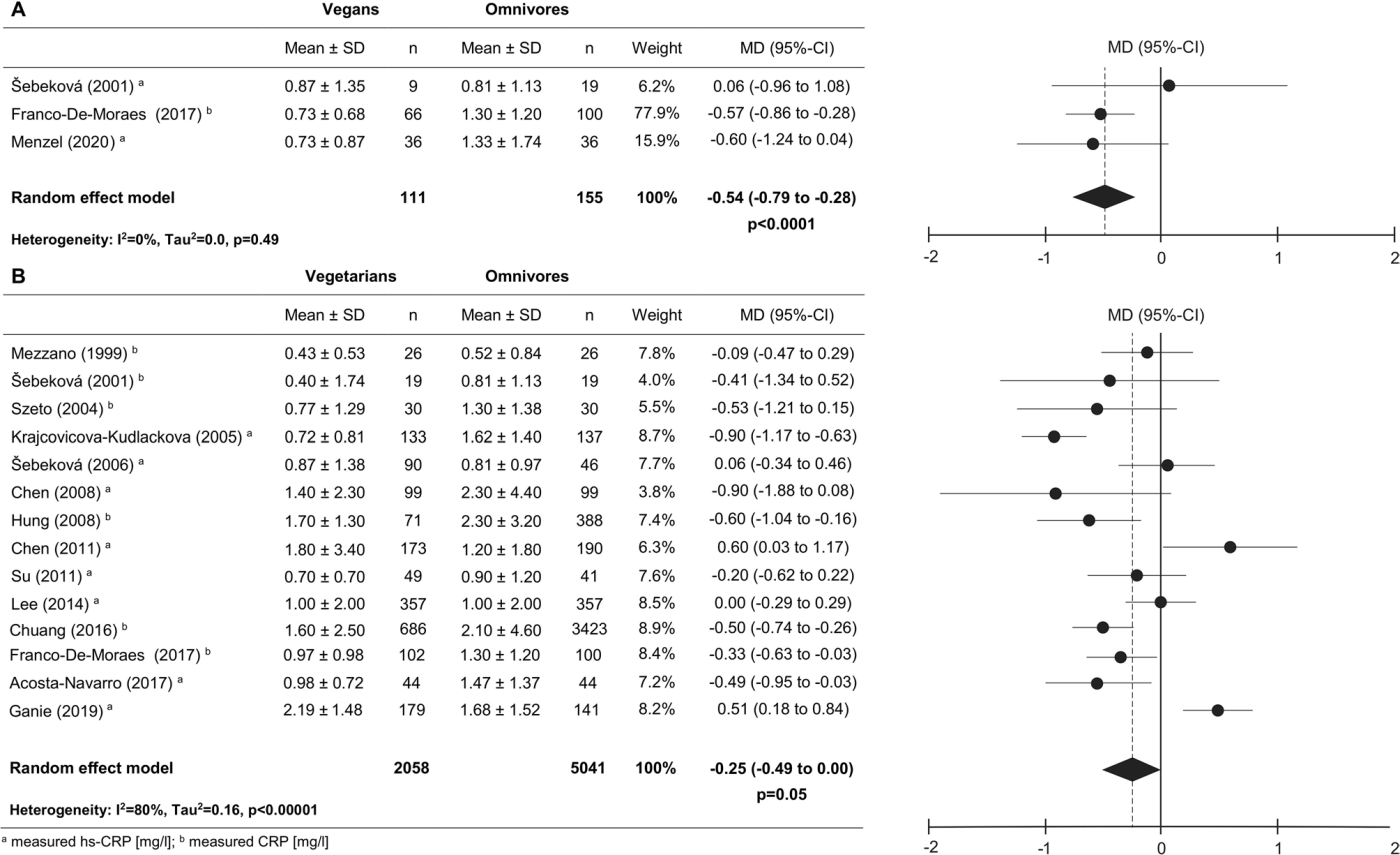
Forest plots of the effect of a vegan and vegetarian diet on CRP concentrations compared to omnivorous diet in apparently healthy participants. Forest plot showing the overall effect of a vegan diet (**A**) or a vegetarian diet (**B**) on CRP concentrations compared to omnivorous diet in apparently healthy participants. Results are presented as mean difference (MD) (95%-CI). The study-specific MD and 95%-CI are represented by the black dot and horizontal line, respectively. The center of the diamond and the vertical dashed line represent the overall effect size of all studies; the width of the diamond represents the overall pooled 95%-CI.

In total, only 3 out of 21 studies investigated the association between a vegan diet and an omnivorous diet in respect to circulating CRP levels in apparently healthy individuals (Fig. 2A, Supplemental Table S1)^18,34,37^. Franco-De-Moraes et al. also investigated the association between a vegan diet and the inflammatory biomarker E-selectin and TNF-ɑ^34^. A recent study provided new data regarding IL-18, IL-1 RA, ICAM-1, adiponectin, omentin-1 and resistin^37^.

When results of three or more studies were available, a meta-analysis has been performed. Hence, in vegans a meta-analysis was only possible for CRP. As depicted in Fig. 2A, the present study observed lower CRP levels in vegans compared to omnivores, showing a mean difference (MD) between vegans and omnivores of − 0.54 mg/l, 95%-CI: − 0.79 to − 0.28 mg/l, *p* < 0.0001). Regarding E-selectin and TNF-ɑ, Franco-De-Moraes et al.^34^ observed no differences between vegans and omnivores (Table 2). Furthermore, Menzel et al.^37^ observed no differences between vegans and omnivores with respect to IL-18, IL-1 RA, ICAM-1, adiponectin, omentin-1 and resistin (Table 2).

**Table 2:** Summary of the concentrations of inflammatory biomarkers in vegetarians, vegan in comparison control group.

References		Study population (n)	*Biomarker values*			*p-value *^*a*^	*p-value *^*b*^
			Vegetarians	Vegans	Omnivores		
**Adiponectin [ng/ml]**							
Ganie et al. (2019)^36^		Apparently healthy (n = 320)	6.75 ± 4.45		7.20 ± 5.38	*0.05*	
Ganie et al. (2019)^36^		PCOS (n = 144)	3.15 ± 2.01		6.33 ± 2.81	< *0.001*	
Menzel et al. (2020)^37^		Apparently healthy (n = 72)		4.76 ± 2.23	4.32 ± 1.57		*0.52*
**E-selectin [pg/ml]**							
Franco-De-Moraes et al. (2017)^34^		Apparently healthy (n = 268)	34.5 ± 20.6	33.2 ± 27.3	39.2 ± 23.5	*0.06*	*0.09*
**ICAM-1 [ng/ml]**							
Menzel et al. (2020)^37^		Apparently healthy (n = 72)		538.9 ± 97.2	569.9 ± 125.0		*0.36*
**IL-6 [pg/ml]**							
Montalcini et al. (2015)^28^		Apparently healthy (n = 52)	1.97 ± 2.80		1.52 ± 1.40	*0.50*	
Lee et al. (2016)^31^		Type 2 diabetes (n = 154)	2.50 ± 1.90		2.00 ± 1.70	*0.04*	
Ganie et al. (2019)^36^		Apparently healthy (n = 320)	8.44 ± 5.64		4.39 ± 3.94	< *0.01*	
Ganie et al. (2019)^36^		PCOS (n = 144)	22.95 ± 14.40		19.86 ± 7.85	*0.91*	
**IL-18 [pg/ml]**							
Menzel et al. (2020)^37^		Apparently healthy (n = 72)		66.1 ± 55.3	89.4 ± 76.3		*0.20*
**IL-1 RA [pg/ml]**							
Menzel et al. (2020)^37^		Apparently healthy (n = 72)		268.5 ± 317.9	231.4 ± 133.3		*0.90*
**MCP-1 [pg/ml]**							
Montalcini et al. (2015)^28^		Apparently healthy (n = 52)	376.6 ± 138.2		320.9 ± 133.7	*0.17*	
**Omentin-1 [ng/ml]**							
Menzel et al.(2020)^37^		Apparently healthy (n = 72)		501.4 ± 163.5	505.0 ± 148.3		*0.92*
**Resistin [ng/ml]**							
Ganie et al. (2019)^36^		Apparently healthy (n = 320)	6.18 ± 3.69		5.48 ± 3.10	*0.16*	
Ganie et al. (2019)^36^		PCOS (n = 144)	10.84 ± 4.54		6.27 ± 2.28	< *0.001*	
Menzel et al. (2020)^37^		Apparently healthy (n = 72)		6.85 ± 1.99	7.20 ± 1.75		*0.43*
**TNF-α [pg/ml]**							
Montalcini et al. (2015)^28^		Apparently healthy (n = 52)	2.41 ± 0.90		2.42 ± 1.10	*0.97*	
Franco-De-Moraes et al. (2017)^34^		Apparently healthy (n = 268)	3.13 ± 2.63	2.67 ± 1.44	3.10 ± 1.96	*0.48*	*0.17*
Ganie et al. (2019)^36^		Apparently healthy (n = 320)	24.18 ± 20.18		22.98 ± 16.42	*0.21*	
Ganie et al. (2019)^36^		PCOS (n = 144)	45.13 ± 35.74		35.47 ± 23.68	*0.91*	

^a^Vegetarian versus omnivore; ^b^Vegan versus omnivore; Values expressed as mean ± SD.

### Association of vegetarian diet and inflammatory biomarkers

19 out of 21 studies^17–27,29–36^ investigated the association between vegetarian diet and CRP, compared to omnivores. In detail, 14 studies^17–25,27,29,33,34,36^ were conducted in apparently healthy participants and six in diseased individuals, i.e. patients with impaired kidney function^26,30,32,35^ , type 2 diabetes^31^ or PCOS^36^. Moreover, in total four studies^28,31,34,36^ investigated differences on E-selectin, TNF-ɑ, IL-6, MCP-1, restistin and adiponectin in vegetarians compared to omnivores (Table 2).

Among the different biomarkers, a meta-analysis was possible for CRP and TNF-ɑ in apparently healthy individuals. Regarding TNF-ɑ no significant differences between vegetarians and omnivores have been observed [mean difference 0.02 pg/ml, 95%-CI: − 0.39 to 0.43 pg/ml, *p* = 0.93]. However, meta-analysis revealed lower CRP levels in vegetarians compared to omnivores, showing a mean difference between vegetarians and omnivores of − 0.25 mg/l (95%-CI: − 0.49 to 0.00 mg/l; *p* = 0.05) (Fig. 2B). The generated funnel plot for CRP showed no evidence for publication bias (Supplemental Figure S1). In addition, also different statistical methods e.g. Egger regression or Funnel plot regression showed no evidence for publication bias (Supplemental Table S2). Interestingly, the association between vegetarian diet and CRP is much more pronounced in pre-diseased individuals with impaired kidney function − 3.91 mg/l (95%-CI: − 5.23 to − 2.60 mg/l; *p* < 0.0001) (Table 3, Supplemental Figure S2). With regard to E-selectin and MCP-1, no significant differences between apparently healthy vegetarians and omnivores have been observed (Table 2). Lower levels of IL-6 have been observed by Ganie et al. (*p* < 0.01)^36^, and in some degree by Montalcini et al. (*p* = 0.50)^28^ in apparently healthy vegetarians compared to omnivores.

**Table 3:** Summarized mean differences showing the overall effect of vegetarian diet on CRP concentrations compared to omnivorous diet in patients with impaired kidney function (A), type 2 diabetes (B) and PCOS (C).

	Vegetarians		Omnivores		Weight	MD (95%-CI)
	Mean ± SD	n	Mean ± SD	n		
**A Impaired kidney function**						
Wu et al. (2011)^26^ ^b^	4.00 ± 0.30	19	8.80 ± 0.40	299	50.4%	− 4.80 (− 4.94 to − 4.66)
Ou et al. (2016)^32^^b^	6.70 ± 9.80	21	6.60 ± 11.2	42	5.3%	0.10 (− 5.29 to 5.49)
Kandouz et al. (2016)^30^^b^	4.53 ± 5.69	16	7.17 ± 8.25	122	13.0%	− 2.64 (− 5.79 to 0.51)
Tseng et al. (2018)^35^^a^	4.00 ± 1.64	15	7.70 ± 7.19	140	31.3%	− 3.70 (− 5.15 to − 2.25)
**Random effect model**		**71**		**603**	**100%**	**− 3.91 (− 5.23 to − 2.60)**
Heterogeneity: I^2^ = 58%, Tau^2^ = 0.89, *p* = 0.07						*p* < *0.0001*
**B Type 2 diabetes**						
Lee et al. (2016)^31^^a^	2.10 ± 2.60	54	1.50 ± 1.90	100		**0.60 (− 0.19 to 1.39)**
						*p* = *0.14*
**C Polycystic Ovary Syndrome**						
Ganie et al. (2019)^36^ ^a^	3.83 ± 1.68	82	2.38 ± 0.88	62		**1.45 (1.03 to 1.87)**
						*p* < *0.0001*

^a^ measured hs-CRP [mg/l] ; ^b^measured CRP [mg/l]; Results are presented as mean difference (MD) (95%-CI).

Regarding pre-diseased participants, Lee et al. observed higher IL-6 levels in participants with type 2 diabetes following a vegetarian diet (*p* = 0.01)^31^. In participants with PCOS no differences were observed (*p* = 0.91)^36^. Furthermore, Ganie et al.^36^ observed lower adiponectin levels in vegetarians compared to omnivores, however, less pronounced in healthy participants (*p* = 0.05) compared to participants with PCOS (*p* < 0.001). With regard to resistin, it has been observed that vegetarians have higher levels compared to omnivores, however significant in patients with PCOS only (*p* < 0.001), not in apparently healthy participants (*p* = 0.16)^36^.

### Sensitivity analysis

Mean difference (95%-CI) of CRP concentrations were performed, according to pre-specified potential sources of heterogeneity of study quality, continent or duration of vegetarian diet. Studies were categorized into high-quality or low-quality studies using a cut-off point of 6 stars. Accordingly, with respect to vegetarian diet, eight studies scored < 6 stars; six studies scored ≥ 6 stars (Table 1, Supplemental Table S3). No relevant changes in results have been observed after stratifying by study quality (*p* for subgroup differences = 0.61, Supplemental Figure S3) or by continent (p for subgroup differences = 0.73, Supplemental Figure S4). Regarding duration of vegetarian diet (< 10 or ≥ 10 years), we observed no difference between both subgroups (p for subgroup differences = 0.35, Supplemental Figure S4). However, we observed lower CRP levels in vegetarians compared to omnivores when the duration of the diet was ≥10 years (-0.42 mg/l (95%-CI: -0.81 to -0.02 mg/l; p=0.04). In contrast, the effect was less pronounced following the diet <10 years (-0.19 mg/l (95%-CI: -0.46 to 0.09 mg/l; p=0.18). In addition, meta-regression reveled no effect modification by BMI (all models *p* > 0.05).

## Discussion

To the best of our knowledge, the present systematic review/ meta-analysis of cross-sectional studies is the most comprehensive evaluation, covering a wide spectrum of inflammatory biomarkers in vegans and vegetarians compared to omnivores, respectively. Accordingly, the present systematic review provides evidence that vegan and vegetarian diets are associated with lower CRP levels, a major marker of inflammation and a mediator of inflammatory processes. Of note, the association was stronger in pre-diseased vegetarians with impaired kidney function. No substantial effects were observed for IL-6, IL-18, IL-1 RA, TNF-ɑ, E-selectin, ICAM-1, MCP-1, adiponectin, omentin-1 and resistin. However, with exception of CRP the most inflammatory biomarkers of interest were investigated only in single studies so far.

Given that CRP is an established biomarker of systemic low-grade inflammation linked to various diseases, e.g. atherosclerotic cardiovascular disease^38^, the results of this review support the suggestion that vegetarian or vegan nutrition habits might ameliorate inflammatory processes and decrease circulating levels of inflammatory biomarkers. These anti-inflammatory properties might reduce risk of chronic inflammatory diseases in vegan or vegetarian populations. Our results are in line with other studies, suggesting an improvement in inflammatory profiles of plant-based/vegetarian-based diets indicated by decreases in CRP levels^14,16^. Moreover, Haghighatdoost et al. found a trend towards lower CRP concentrations in subjects following a vegetarian diet for at least 2 years^3^, while no significant effect was found in studies using a minimum duration of 6 months of vegetarianism. In line with these observations, the present study revealed that the association might depend on the duration of the diet. According to the sensitivity analysis, the effect was more obvious in participants following a vegetarian diet of at least 10 years. Taken together, our findings provide evidence that a vegan or vegetarian diet may be beneficial to prevent or counteract inflammatory state underlying numerous chronic diseases and therefore might be a nutritional approach to reduce risk of chronic diseases. Moreover, adoption of a vegan or vegetarian diet may also have beneficial effects in pre-diseased populations. Next to the decreased level of CRP in vegetarian patients with impaired kidney function, a plant-based diet may hamper the development or progression of some complications of chronic kidney disease, due to the associated cardioprotective, anti-oxidant, and lipid-lowering properties^39^. Current evidence proposed this also for other cardiometabolic diseases, as vegetarian and vegan diets present potential advantages in managing type 2 diabetes offering additional benefits for the comorbidities of cardiovascular disease, kidney disease, and neuropathy^40^.

Up to date, the mechanisms by which vegan or vegetarian diets might reduce the low-grade inflammatory state remain underestimated, but research holds great promise in revealing the mechanisms linking dietary patterns with inflammation. Accordingly, it has been suggested that exposure to animal foods may favor an intestinal environment which could trigger systemic inflammation^34^. Indeed some studies noticed differences in gut microbiota composition of vegans/vegetarian compared to omnivores, who differ according to their inflammatory and metabolic profiles^34,41^. However, a recent systematic review from 2019 noticed no consistent association between a vegan or vegetarian diet and microbiota composition compared to omnivores^42^. Interestingly, recent studies revealed the role in regulating of gut microbiota and gut homeostasis by inflammasomes^43^. These represent a group of protein complexes that recognize a diverse set of inflammation-inducing stimuli and promote the secretion of pro-inflammatory cytokines^44^. Those mechanisms may affect the immune homeostasis related to coinciding decrease in the future-risk for metabolic diseases, e.g. metabolic syndrome or atherosclerosis^44^. Although further research is clearly required, the role of inflammasomes in regulating of gut flora represents a new promising research field which may help elucidate the mechanisms by which diet impacts gut microflora, inflammation and health^41^.

Despite 21 studies have been identified for inclusion in this review, many inflammatory biomarkers of interest were not investigated upon or were only explored in single studies. Thus, the present meta-analysis could not provide comprehensive conclusion about the associations between a vegan or vegetarian diet and each inflammatory marker. Therefore, more research is highly warranted to evaluate associations between a vegan or vegetarian diet and inflammatory biomarkers.

The present systematic review/meta-analysis has several strengths. Our study covered a comprehensive spectrum of biomarkers that reflect chronic inflammation and immune reactions, including a set of novel molecules at the site of adipose-tissue induced inflammatory response. Importantly, we focused on comprehensive nutritional vegan or vegetarian habits rather than on the use of single dietary supplements or artificial dietary approaches, which allows translation of the findings to general populations. In comparison to other meta-analyses^3,16^, our study comprises no mixture of vegetarian and vegan diet, but performed strict separate analyses regarding vegans and vegetarians. Moreover, we investigated the association between biomarker profiles in apparently healthy and diseased patients, respectively.

Some limitations of our study deserve to be mentioned. RCTs are considered as the gold standard for establishing causal conclusions, however, published RCTs of vegan or vegetarian diets on inflammatory biomarkers based on our inclusion criteria are missing. Therefore, the present systematic review/ meta-analysis could include cross-sectional studies only, which does not allow for causal inference. The majority of the studies included a low number of participants. Furthermore, high heterogeneity among the studies in vegetarians was detected. It should be noted that our analysis is restricted by the data provided within the available studies each having its own methodological characteristics and potential drawbacks. In this respect, we should acknowledge the differences in the assay quality measurements and selection of investigated inflammatory biomarkers.

In conclusion, this systematic review and meta-analysis provide evidence that either vegan or vegetarian diet is associated with lower CRP concentrations compared to omnivores in apparently healthy participants and metabolically afflicted patients. Further research is highly warranted, as several biomarkers of interest were only investigated in single studies so far.

## Methods

### Study protocol

The study was planned, conducted and reported according to the recommendations of the Preferred Reporting Items for Systematic Reviews and Meta-Analyses (PRISMA) statement. The study protocol was prospectively registered with the International Prospective Register of Systematic Reviews (PROSPERO; registration number CRD42018079220).

### Data sources and search strategy

A systematic literature search was conducted to identify relevant articles using PubMed and Embase databases until 15th of April 2020. No restriction was made in terms of language or the date of study publications. The following search terms were used (MeSH term or text words): (‘vegan’, ‘vegans’, ‘veganism’ OR ‘vegetarian’, ‘vegetarians’, ‘vegetarianism’) AND (‘adiponectin’ OR ‘c-reactive protein’, ‘CRP’, ‘hscrp’, OR ‘e-selectin’ OR ‘intercellular adhesion molecule-1’, ‘ICAM-1’ OR ‘interleukin-1 receptor antagonist’, ‘IL-1RA’ OR ‘interleukin-6’, ‘IL-6’ OR ‘interleukin-18’, ‘IL-18’ OR ‘monocyte chemoattractant protein-1’, ‘MCP-1’ OR ‘intelectin-1’, ‘omentin-1’ OR ‘resistin’ OR ‘TNF-alpha’, ‘TNF-α’). Additionally, reference lists of related original articles, review articles and meta-analyses were further screened for potentially eligible publications using a manual approach. When necessary, the relevant authors were contacted by the investigators to acquire important missing information; in case of non-respondents, studies were excluded.

### Study selection and eligibility criteria

Studies were eligible for inclusion in the review if they reported results of vegetarianism or veganism, in comparison to a control group on circulating levels of inflammatory biomarkers in adults (aged ≥ 18 years). In detail, vegetarian diets were defined as meat, poultry, fish abstinence, and the partial exclusion of animal products such as eggs, and/or dairy products. Vegan diet implies the complete exclusion of any animal products (consumption less than one meal per month). Of note, one study mentioned participants as “strict vegetarian” which are considered as vegans in the present study^34^. Participants of the control groups were considered if they eat meat products (omnivorous western diet). The present review included all study designs, i.e. cross sectional studies, prospective cohort studies and RCT. Moreover, no specific criterion was considered for the duration of being on a vegan/vegetarian diet, except for RCT (an intervention time of at least 4 weeks was considered). Only studies published in English or German have been included in the present study. With regards to outcome assessment, our initial approach was to evaluate the existing literature on the well-known biomarkers of inflammation CRP, IL-6, IL-18, IL-1 RA, TNF-ɑ, E-selectin, ICAM-1, MCP-1, adiponectin, omentin-1 and resistin. Studies were excluded if they assessed the effects of a general healthy lifestyle that included a vegan/vegetarian diet only as one component. Further, studies investigating a modified vegan or vegetarian diet (eg. raw, low-fat, low-carbohydrate, low-calorie, low-protein, gluten-free) were excluded. Two reviewers (JM and AJ) independently reviewed the titles and abstracts of all potential studies, followed by full-text selection. Disagreement was resolved by discussion and consensus, and if needed the opinion of a third author (CW) was decisive.

### Data extraction and quality assessment

For each of the selected studies, the following information was recorded in the data extraction sheets. Study characteristics were extracted including first author’s last name, publication year, country, study design, sample size, mean age, gender distribution (proportion of males), duration of being on a vegan/vegetarian diet, and inflammatory biomarker values for vegans/vegetarians and omnivores (mean ± SD). Proportion of males was calculated when not provided. If necessary, biomarker values have been transformed to mean and standard deviation (SD). For studies conducted in a diseased population, the disease was also extracted. If studies introduced additional interventions after a certain period of time, only values from the dietary intervention were extracted. Extracted data were converted to international units. As the inflammatory biomarker CRP has been measured as CRP or hsCRP in different studies, the manuscript refer both terms as CRP. Two reviewers evaluated study quality independently using the Newcastle—Ottawa Quality Assessment Scale adapted for cross-sectional studies^45^; if needed, the opinion of a third author (CW) was decisive.

### Meta-analysis

To estimate the pooled effect of vegan and vegetarian diets compared to omnivorous diet on each respective inflammatory biomarker, the effect size was calculated using the differences between means (95%-CI) of inflammatory biomarkers concentrations (vegans vs. omnivores; vegetarians vs. omnivores), separated by health status (apparently healthy or diseased). Meta-analysis have been planned to perform when at least results of 3 studies were available. Summary estimates and corresponding 95%-CI were derived using a random-effects model, which assigns a weight to each study on the basis of an individual study’s inverse variance. Consistency of results was evaluated based on calculation of I^2^ index, also known as a ‘heterogeneity index’, which examines the null hypothesis that all studies are evaluating the same effect. To identify publication bias, a funnel plot was generated and examined; asymmetry was assessed by visual inspection. Further, publication bias was analyzed through Egger’s regression, Begg rank correlation, funnel plot regression, and trim-and-fill tests using an available macro PubBias for SAS^46^. This was exclusively applied to meta-analyses with ≥ 10 studies. Last, sensitivity analysis for study quality was performed to investigate single studies as potential sources of heterogeneity including study quality score (< 6 or ≥ 6 stars), continents (Asia, Europe, South America) and duration of vegetarian diet (< 10 or ≥ 10 years). Of note, for Hung et al.^23^ and Ganie et al.^36^ the last classification was not possible, due to missing information on duration of vegetarian diet. Meta‐regression analyses were performed examine whether there were differences in inflammatory biomarkers between dietary groups, controlling for BMI (mean BMI or difference of BMI between diet groups). These analyses were performed whenever the number of studies was sufficient (n > 10). These analyses were performed using statistical software R (version 4.0.2) with the Meta‐package. Other statistical analyses were performed using statistical software Review Manager (RevMan), Version 5.3. (Copenhagen: The Nordic Cochrane Centre, the Cochrane Collaboration, 2014) and SAS software, version 9.4 (SAS institute, Cary, N.C., USA). *p* values of < 0.05 were considered statistically significant. All modifications of figures were performed using Adobe Illustrator CC 2019.

## Supplementary information

Supplementary Information.
