# Supplementary material for: Systematic review and meta-analysis of the associations of vegan and vegetarian diets with inflammatory biomarkers
Source: Sci Rep. 2020 Dec 10;10:21736. doi: 10.1038/s41598-020-78426-8 (PMC7730154; doi:10.1038/s41598-020-78426-8)
Supplement: Supplementary file 1 — Supplementary Information. [file 41598_2020_78426_MOESM1_ESM.docx]

**Systematic review and meta-analysis of the associations of vegan and vegetarian diets with inflammatory biomarkers**

Juliane Menzel, Afraa Jabakhanji, Ronald Biemann, Knut Mai, Klaus Abraham, Cornelia Weikert


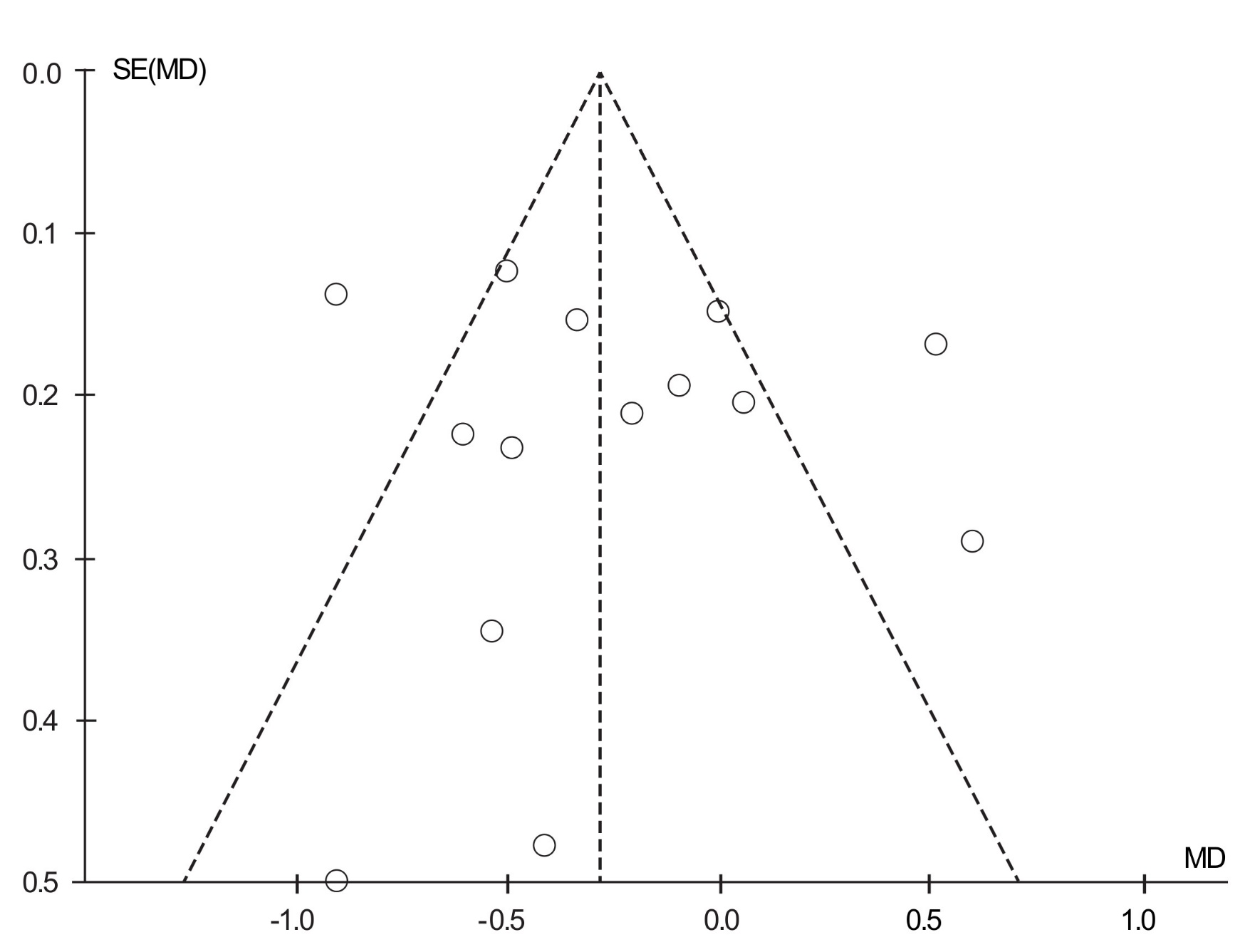
**Supplemental Figure S1.** Funnel plot for studies investigating the association between CRP levels and a vegetarian diet.

**Supplemental Table S1.** Summary of the CRP concentrations in vegetarians, vegans in comparison to control group

|  |  |  | **Biomarker values** | | |  |  |
| --- | --- | --- | --- | --- | --- | --- | --- |
| **Author** | **Year** | **Study population (n)** | **Vegetarian** | **Vegan** | **Omnivores** | *p-value ^a^* | *p-value ^b^* |
| Mezzano et al. ^d^ | 1990 | Apparently healthy (n=52) | 0.43 ± 0.53 |  | 0.52 ± 0.84 | *>0.05* |  |
| Šebeková et al. ^d^ | 2001 | Apparently healthy (n=61) | 0.40 ± 1.74 | 0.87 ± 1.35 | 0.81 ± 1.13 | *>0.05* | *>0.05* |
| Szeto et al. ^d^ | 2004 | Apparently healthy (n=60) | 0.77 ± 1.29 |  | 1.30 ± 1.38 | *<0.01* |  |
| Krajcovicova-K. et al. ^c^ | 2005 | Apparently healthy (n=270) | 0.72 ± 0.81 |  | 1.62 ± 1.40 | *<0.001* |  |
| Šebeková et al.^c^ | 2006 | Apparently healthy (n=136) | 0.87 ± 1.38 |  | 0.81 ± 0.97 | *>0.05* |  |
| Chen et al. ^c^ | 2008 | Apparently healthy (n=198) | 1.40 ± 2.30 |  | 2.30 ± 4.40 | *0.03* |  |
| Hung et al.^d^ | 2008 | Apparently healthy (n=459) ^e^ | 1.70 ± 1.30 |  | 2.30 ± 3.20 | *0.003* |  |
| Chen et al.^c^ | 2011 | Apparently healthy (n=363) | 1.80 ± 3.40 |  | 1.20 ± 1.80 | *0.05* |  |
| Su et al. ^c^ | 2011 | Apparently healthy (n=90) | 0.70 ± 0.70 |  | 0.90 ± 1.20 | *0.44* |  |
| Wu et al.^c^ | 2011 | Haemodialysis patients (n=318) | 4.00 ± 0.30 |  | 8.80 ± 0.40 | *<0.05* |  |
| Lee et al. ^c^ | 2014 | Apparently healthy (n=714) ^e^ | 1.00 ± 2.00 |  | 1.00 ± 2.00 | *0.74* |  |
| Chuang et el. ^d^ | 2016 | Apparently healthy (n=4109) | 1.60 ± 2.50 |  | 2.10 ± 4.60 | *0.06* |  |
| Kandouz et al. ^d^ | 2016 | Kidney failure (n=138) | 4.53 ± 5.69 |  | 7.17 ± 8.25 | *0.17* |  |
| Lee et al. ^c^ | 2016 | Type 2 diabetes (n=154) | 2.10 ± 2.60 |  | 1.50 ± 1.90 | *0.01* |  |
| Ou et al. ^c^ | 2016 | Dialysis Patients (n=63) | 6.70 ± 9.80 |  | 6.60 ± 11.2 | *0.35* |  |
| Acosta-Navarro et al. ^c^ | 2017 | Apparently healthy (n=88) | 0.98 ± 0.72 |  | 1.47 ± 1.37 | *0.08* |  |
| Franco-De-Moraes et al. ^d^ | 2017 | Apparently healthy (n=268) | 0.97 ± 0.98 | 0.73 ± 0.68 | 1.30 ± 1.20 | *0.05* | *0.002* |
| Tseng et al. ^c^ | 2018 | Haemodialysis patients (n=155) | 4.00 ± 1.64 |  | 7.70 ± 7.19 | *0.02* |  |
| Ganie et al. ^c^ | 2019 | Apparently healthy (n=320) | 2.19 ± 1.48 |  | 1.68 ± 1.52 | *<0.01* |  |
| Ganie et al. ^c^ | 2019 | PCOS (n=144) | 3.83 ± 1.68 |  | 2.38 ± 0.88 | *0.01* |  |
| Menzel et al.^c^ | 2020 | Apparently healthy (n=72) |  | 0.73 ± 0.87 | 1.33 ± 1.74 |  | *0.25* |

^a^ Vegetarian versus omnivore; ^b^ Vegan versus omnivore; ^c^ measured hs-CRP [mg/l]; ^d^ measured CRP [mg/l]; all values expressed as mean ± SD; ^e^ less than 20% patients with metabolic syndrome

**Supplemental Table S2:** Statistical tests of Publication Bias

| **Method** | **p-Value** |
| --- | --- |
| **Egger Regression** | 0.86 |
| **Begg Rank Correlation** |  |
| Prediction: Variance | 0.87 |
| Prediction: Sample Size | 0.95 |
| **Funnel Plot Regression** | 0.67 |
| **Method** | **Publication Bias Present** |
| **Trim and Fill** |  |
| Right Tail | No |
| Left Tail | No |
| Both Tail | No |

The macro PubBias for SAS ^1^ was used.

1 Rendina-Gobioff, G. & Kromrey, J. PUB_BIAS: A SAS® Macro for Detecting Publication Bias in Meta-Analysis. University of South Florida, Tampa, FL. https://analytics.ncsu.edu/sesug/2006/PO04_06.PDF (Accessed September 2004,2020) (2006).

**Supplemental Figure S2.** Forest plots of the effect of vegetarian diet on CRP concentrations compared to omnivorous diet in pre-diseased participants.

**
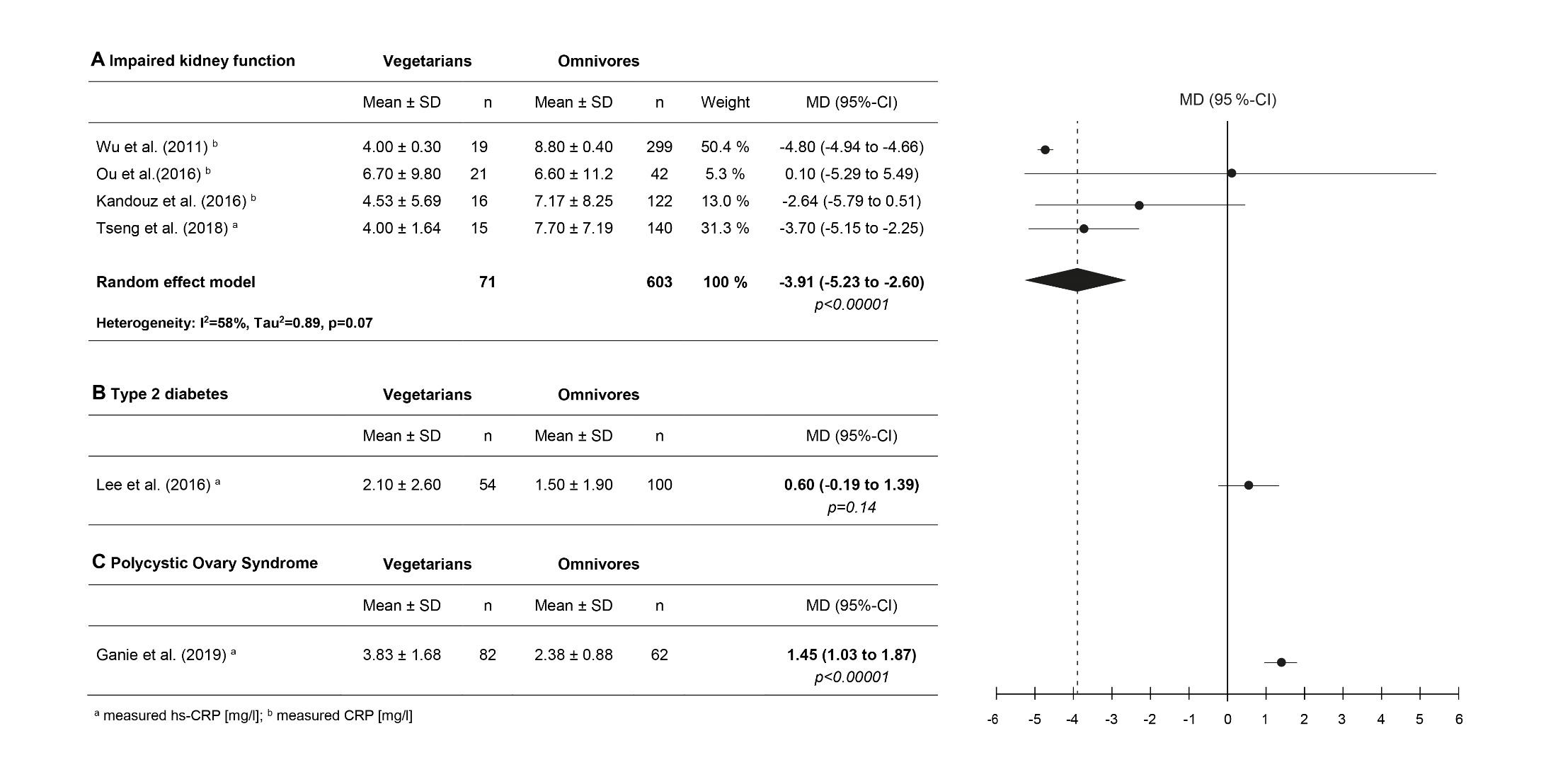
**

Forest plot showing the overall effect of a vegetarian diet on CRP concentrations compared to omnivorous diet in participants with impaired kidney function (A), with type 2 diabetes (B) or with polycystic ovary syndrome (C). Results are presented as mean difference (MD) (95%-CI). The study-specific MD and 95%-CI are represented by the black dot and horizontal line, respectively. The center of the diamond and the vertical dashed line represent the overall effect size of all studies; the width of the diamond represents the overall pooled 95%-CI.

**Supplemental Table S3:** Quality of included studies in the systematic review/ meta-analysis

|  | **Selection** | | | | **Comparability** | **Outcome** | | **Total** |
| --- | --- | --- | --- | --- | --- | --- | --- | --- |
|  | Maximal 5 ★ | | | | Maximal 2 ★ | Maximal 3 ★ | | Maximal 10 ★ |
| **Author (Year)** | **REP** | **SAM** | **NREP** | **AE** | **COM** | **ASS** | **STAT** |  |
| Mezzano (1999) | **-** | **-** | **-** | **★★** | **-** | **★★** | **★** | 5 |
| Šebeková (2001) | **-** |  | **-** | **★★** | **-** | **★★** | ★ | 5 |
| Szeto (2004) | **-** | **-** | **-** | **-** | **-** | **★★** | ★ | 3 |
| Krajcovicova-Kudlackova (2005) | **★** | **★** | **-** | **-** | **★** | **★★** | ★ | 6 |
| Šebeková (2006) | **-** | **-** | **-** | **★★** | **-** | **★★** | ★ | 5 |
| Chen (2008) | **★** | **★** | **-** | **-** | **-** | **★★** | ★ | 5 |
| Hung (2008) | **★** | **★** | **-** | **-** | **-** | **★★** | ★ | 5 |
| Chen (2011) | **★** | **★** | **-** | **-** | **★** | **★★** | ★ | 6 |
| Su (2011) | **-** | **-** | **-** | **-** | **-** | **★★** | ★ | 3 |
| Wu (2011) | **-** | **-** | **-** | **★★** | **-** | **★★** | ★ | 5 |
| Lee (2014) | **★** | **★** | **-** | **★** | **-** | **★★** | ★ | 6 |
| Montalcini (2015) | **-** | **-** | **-** | **★★** | **-** | **★★** | ★ | 5 |
| Chuang (2016) | **★** | **★** | **-** | **★★** | **-** | **★★** | ★ | 7 |
| Kandouz (2016) | **★** | **-** | **-** | **★** | **-** | **★★** | ★ | 5 |
| Lee (2016) | **-** | **-** | **-** | **★** | **-** | **★★** | ★ | 4 |
| Ou (2016) | **-** | **-** | **-** | **★** | **-** | **★★** | ★ | 4 |
| Acosta-Navarro (2017) | **★** | **-** | **-** | **★★** | **-** | **★★** | ★ | 6 |
| Franco-De-Moraes (2017) | **★** | **★** | **-** |  | **-** | **★★** | ★ | 5 |
| Tseng (2018) |  | **★** | **-** | **★** | **-** | **★★** | ★ | 5 |
| Ganie (2019) | **★** | **★** | **-** | **★★** | **-** | **★★** | ★ | 7 |
| Menzel (2020 | **-** | **-** | **-** | **★★** | **★** | **★★** | ★ | 6 |

Two reviewers evaluated study quality independently using the Newcastle - Ottawa Quality Assessment Scale adapted for cross-sectional studies. Abbreviations: REP =Representative of the sample. SAM = Sample size. NREP = Non-respondents. AE= Ascertainment of the exposure (risk factor). COM= The subjects in different outcome groups are comparable ASS= Assessment of the outcome. STAT =Statistical tests.

**Supplemental Figure S3.** Forest plots of the effect of a vegetarian diet on CRP concentrations compared to omnivorous diet in apparently healthy participants according to study quality


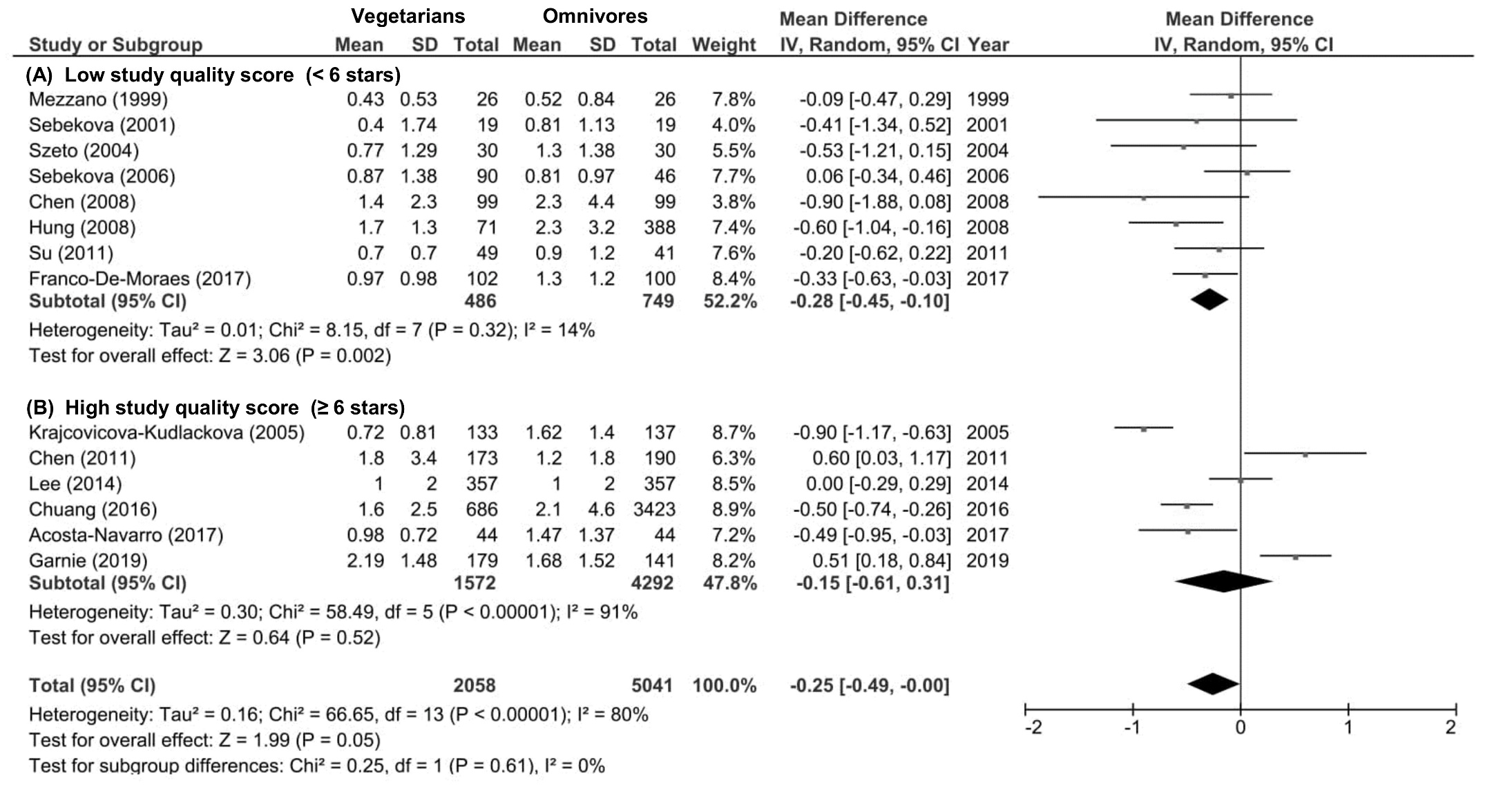


Forest plot showing the overall effect of a vegetarian diet on CRP concentrations compared to omnivorous diet in apparently healthy participants according to studies with a low study quality score (< 6 stars) (A) or according to studies with a high study quality score (≥ 6 stars) (B). Results are presented as mean difference (MD) (95%-CI). The study-specific MD and 95%-CI are represented by the black dot and horizontal line, respectively. The center of the diamond and the vertical dashed line represent the overall effect size of all studies; the width of the diamond represents the overall pooled 95%-CI.

**Supplemental Figure S4**. Forest plots of the effect of a vegetarian diet on CRP concentrations compared to omnivorous diet in apparently healthy participants according to continent


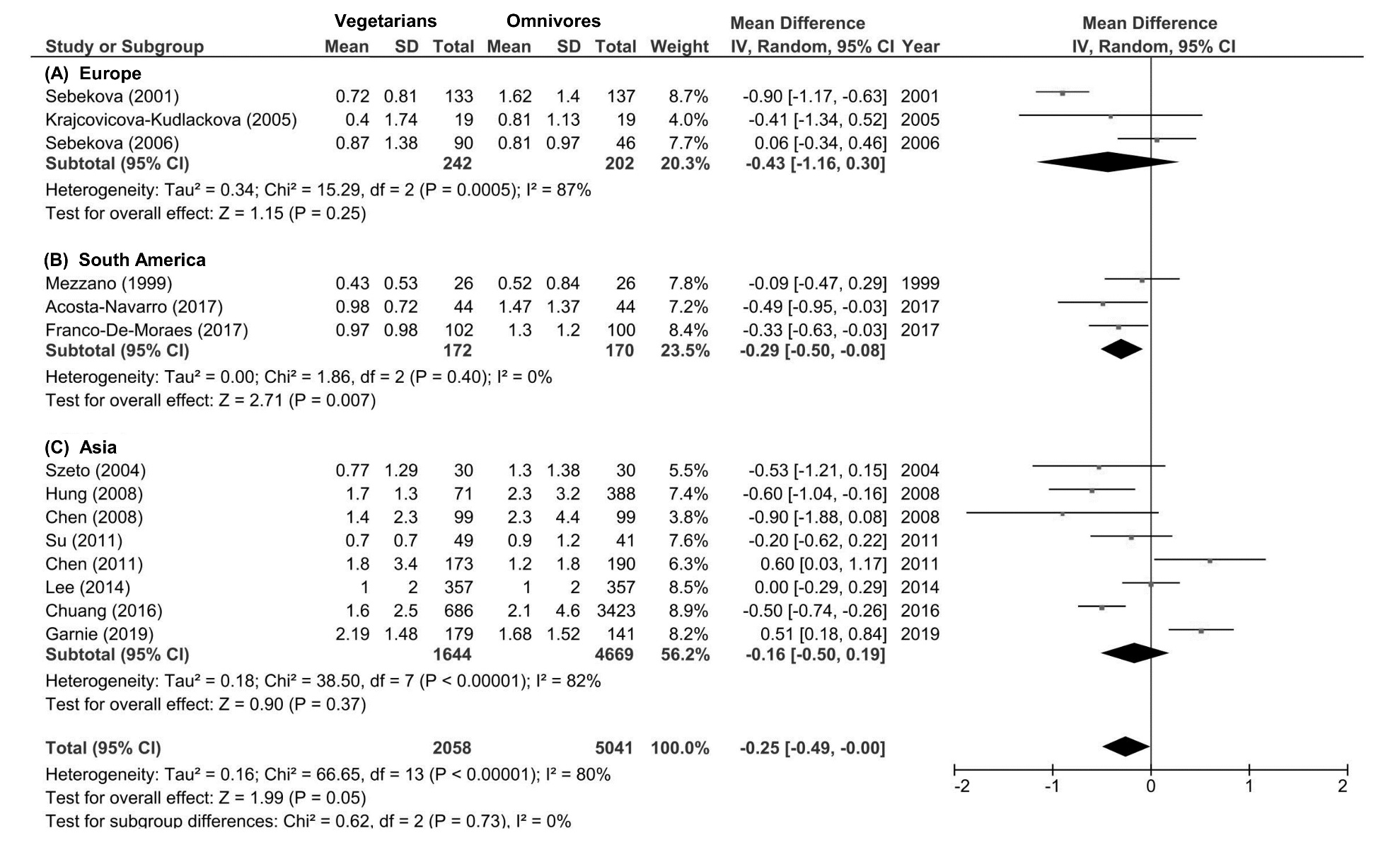


Forest plot showing the overall effect of a vegetarian diet on CRP concentrations compared to omnivorous diet in apparently healthy participants according to studies conducted in Europe (A), South America (B) or Asia (C). Results are presented as mean difference (MD) (95%-CI). The study-specific MD and 95%-CI are represented by the black dot and horizontal line, respectively. The center of the diamond and the vertical dashed line represent the overall effect size of all studies; the width of the diamond represents the overall pooled 95%-CI.

**Supplemental Figure S5.** Forest plots of the effect of a vegetarian diet on CRP concentrations compared to omnivorous diet in apparently healthy participants according to duration of vegetarian diet


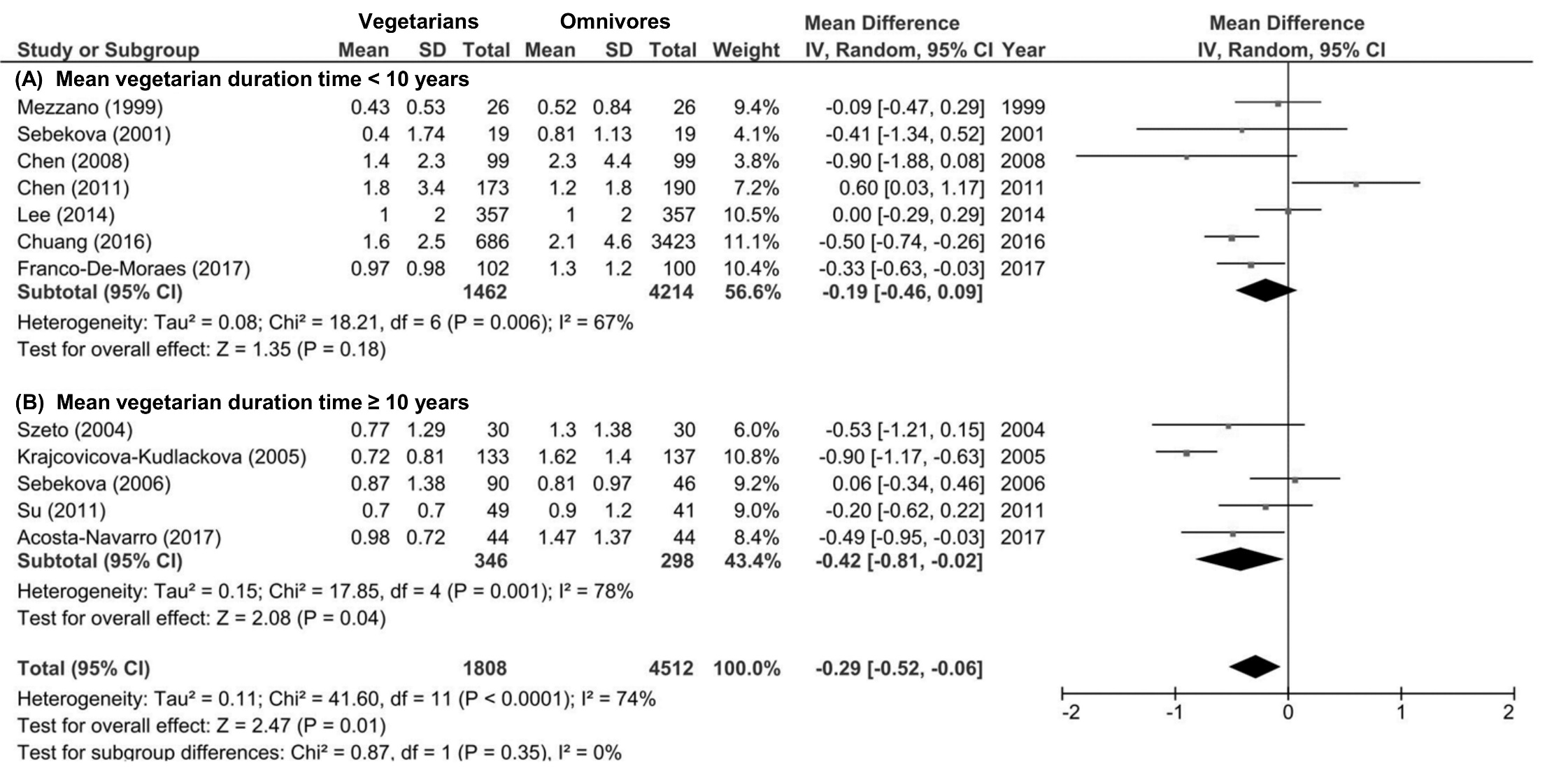


Forest plot showing the overall effect of a vegetarian diet on CRP concentrations compared to omnivorous diet in apparently healthy participants according to studies with participants following a vegetarian diet < 10 years (A) or ≥ 10 years (B). Results are presented as mean difference (MD) (95%-CI). The study-specific MD and 95%-CI are represented by the black dot and horizontal line, respectively. The center of the diamond and the vertical dashed line represent the overall effect size of all studies; the width of the diamond represents the overall pooled 95%-CI.
